# Phages as a Cohesive Prophylactic and Therapeutic Approach in Aquaculture Systems

**DOI:** 10.3390/antibiotics9090564

**Published:** 2020-09-01

**Authors:** Maciej Żaczek, Beata Weber-Dąbrowska, Andrzej Górski

**Affiliations:** 1 Bacteriophage Laboratory, Ludwik Hirszfeld Institute of Immunology and Experimental Therapy, Polish Academy of Sciences, 53-114 Wrocław, Poland; weber@hirszfeld.pl (B.W.-D.); andrzej.gorski@hirszfeld.pl (A.G.); 2 Phage Therapy Unit, Ludwik Hirszfeld Institute of Immunology and Experimental Therapy, Polish Academy of Sciences, 53-114 Wrocław, Poland; 3 Infant Jesus Teaching Hospital, Medical University of Warsaw, 02-005 Warsaw, Poland

**Keywords:** phage therapy, aquacultures, fish industry, seafood industry, bacterial disease, fish spoilage, antibiotic resistance, animal infections

## Abstract

Facing antibiotic resistance has provoked a continuously growing focus on phage therapy. Although the greatest emphasis has always been placed on phage treatment in humans, behind phage application lies a complex approach that can be usefully adopted by the food industry, from hatcheries and croplands to ready-to-eat products. Such diverse businesses require an efficient method for combating highly pathogenic bacteria since antibiotic resistance concerns every aspect of human life. Despite the vast abundance of phages on Earth, the aquatic environment has been considered their most natural habitat. Water favors multidirectional Brownian motion and increases the possibility of contact between phage particles and their bacterial hosts. As the global production of aquatic organisms has rapidly grown over the past decades, phage treatment of bacterial infections seems to be an obvious and promising solution in this market sector. Pathogenic bacteria, such as *Aeromonas* and *Vibrio*, have already proved to be responsible for mass mortalities in aquatic systems, resulting in economic losses. The main objective of this work is to summarize, from a scientific and industry perspective, the recent data regarding phage application in the form of targeted probiotics and therapeutic agents in aquaculture niches.

## 1. Introduction

Fisheries and the aquaculture sector are key elements in the food industry that supply food to every part of the world. According to the Food and Agriculture Organization of the United Nations (FAO), global fish production reached 179 million tons in 2018, nearly 90% of which has been consumed by humans [1]. The numbers speak for themselves. In the past 30 years, global aquaculture production increased by over 500% and fish consumption by 122% [1]. In the United States alone, the estimated freshwater and marine aquaculture production reached a value of USD1.45 billion in 2016, an increase of over USD60 million from 2015 [2]. On a global scale, China is the largest producer of seafood products in the world, followed by India, Indonesia, Vietnam, and Bangladesh. The most commonly farmed species in the fish industry are carps, tilapias, and salmon. In addition to fish species, aquaculture production consists of, among others, clams, oysters, and shrimp [2].

Fish constitute crucial nutrition in many areas. It is not only one of the healthiest foods on Earth but also one of the least impactful on the natural environment. Seafood is rich in protein, vitamins, and minerals. The most recognized nutritional benefits come from the omega-3 fatty acids DHA (docosahexaenoic acid) and EPA (eicosapentaenoic acid), which might help in the prevention or mitigation of common cardiovascular chronic diseases and have been recently associated with fetal development (including neuronal, retinal, and immune functions) and Alzheimer’s disease [3]. The Dietary Guidelines for Americans recommend that adults consume about eight ounces/approx. 230 g per week of a variety of seafood, including at least some choices higher in the omega-3 fatty acids EPA and DHA [2]. The tight bonds between turnover in the fish industry and the amount of fish consumed have been exposed by the recent COVID-19 pandemic outbreak, which halted sales in restaurants. For instance, weekly sales at Portland Fish Exchange shrunk to less than a third of prepandemic levels [4].

The food industry is continuously challenged with the threat of microbial contamination, and aquatic hatcheries are no exception. While only a few antibiotics are approved for aquaculture, some of them (e.g., tetracyclines and oxolinic acid) are in regular use in Chile and Europe, respectively, in the fish industry [5]. The overuse of antibiotics has further escalated this problem, resulting in the increasing emergence of antibiotic-resistant foodborne pathogens [6]. This impact has been extensively documented both in the laboratory and in the field [5]. Antibiotics such as sulfonamides and tetracyclines have been approved in the United States not only for disease prevention and treatment in aquacultures but also for growth promotion in catfish, trout, salmon, and lobster [7]. In China, a total of 20 antibiotics belonging to eight categories have been reported in use. However, only 13 antibiotics have been authorized for application in Chinese aquaculture, and another 12 antibiotics used are not authorized [8]. Maintenance of the seafood industry, a diverse and global branch of the world economy, raises obvious concerns regarding possibly significant financial losses and the risk of insufficient food supply in some parts of the world. The most important aim is to deliver fresh products to the market, free of any sort of spoilage and/or contamination. Currently, increasing global demand for seafood can only be met through intensive aquaculture production [9].

In conjunction with the above, Southeast Asia dominates in aquaculture production. This is mirrored in the number of articles on phage characterization and application in aquatic ecosystems of an Asian origin [10,11,12,13,14,15,16]. Outside of Asia, Chilean salmonid farms are one of the most numerous in the fish industry. Over recent years, there has been a consistent increase in the amount of antimicrobials used by Chilean salmonid farms, from 143.2 tons in 2010 to 382.5 tons in 2016. Notably, until 2015, the use of antibiotics in Chilean aquacultures was higher than the amount reported [17].

## 2. Bacterial Disease and Spoilage in the Fish and Seafood Industry

Due to expanding urbanization and competition in the fish industry, there is a growing demand for safer and better quality products [18]. No one needs to be convinced that fish spoilage is a common phenomenon. FAO concludes that currently, half of all fish eaten by people globally are farm-raised [19]. As waterborne pathogens can spread more easily and at faster rates than in terrestrial systems, fish farms are at constant risk of pathogen outbreaks [20]. Water is a natural reservoir for countless species of microorganisms and acts as a carrier for pathogens. Even live feed organisms like *Artemia* are able to accumulate bacterial pathogens from the surrounding water and transfer them into aquacultures during fish feeding [21]. Such a complex and dynamic selection of bacteria may be challenging in terms of isolation, identification, and, finally, annihilation. One need only mention that known genome sizes of bacteria range from under 0.6 to 10 megabases.

The practice of stocking and growing fish at very high densities in closed recirculating aquaculture systems has led to the emergence of several bacterial pathogens. Cumulative mortality rates in young fish can reach 75% in a matter of weeks [7]. Phage treatment as a natural remedy against bacterial infections in the food industry has been reported in numerous peer-reviewed articles and books within the past decades [22,23,24,25,26,27], with the first work published as early as in the 1930s [28]. Phage products intended for use in the food industry are already commercially available and have been proven to be useful in protecting a vast range of food and crops [24,29,30,31,32].

Control and treatment options for a vast range of fish pathogens using vaccines and drugs are either inadequate, inefficient, or impracticable [33]. Furthermore, seafood is more susceptible to microbial spoilage than meat and has a relatively short shelf-life. There are several reasons for the differences between red meat and fish. First of all, red meat has a lower pH and is less moist. Postmortem pH in fish flesh increases due to the utilization of large amounts of amino acids and other low molecular weight compounds by fish spoilage bacteria [34]. In addition, contamination of seafood may originate not only from water but also from soil [35]. Microorganisms can be found on all the outer surfaces (skin and gills) and in the intestines of live and newly caught fish. It has been noted that the warmer the natural waters are, the higher the bacterial count associated with fish [18]. Global warming, which is on the rise, will certainly challenge the entire industry in terms of eliminating bacterial pathogens both in the water as well as during the food processing steps in the upcoming decades. Moreover, global warming may shift the diseases traditionally confined to warm subtropical geographical areas, such as shellfish-related gastroenteritis caused by *Vibrio parahaemolyticus* [36], northwards.

The three most common bacterial genera identified as fish pathogens are *Aeromonas*, *Flavobacterium*, and *Vibrio*, followed by *Edwardsiella*, *Yersinia*, *Renibacterium*, *Streptococcus*, and *Mycobacteria* [33]. Examples of pathogenic bacterial flora associated with live fish, as well as fish muscle (meat) intended for consumption, are presented in Table 1.

Notably, fish pathogens can freely invade the flesh of dead fish by moving between the muscle fibers [18]. Fish and seafood spoilage is inevitably associated with the formation of chemical compounds that can be toxic for humans, such as trimethylamine, followed by ammonia, H_2_S, and indole [35]. There are numerous different bacteria capable of producing biogenic amines that have been isolated from fish muscle [37]. Even fish products with high salt content may spoil due to the growth of halophilic bacteria or anaerobic bacteria [38]. Although fish spoilage is usually easy to detect by observing changes in color, smell, or tenderness of fish flesh, many water-associated pathogens are opportunistic and may remain undetected in living organisms [9]. In such a way, fish and seafood pathogens transferred to the human body can cause septicemia. For instance, marine *Vibrio* bacteria (in particular, *V. vulnificus* and *V. parahaemolyticus*) can be found on oysters, a popular seafood that is consumed raw. This model of consumption puts people at great risk. It is estimated that in the USA, approximately 84,000 people contract a foodborne infection from *Vibrio spp.* every year, with symptoms such as diarrhea, along with abdominal cramps, nausea, vomiting, headache, chills, and low-grade fever; fatal cases have been reported as well [39]. *V. vulnificus* can also be responsible for chronic liver disease accompanied by high mortality [40]. Analysis of 117 samples of blue mussels, seawater, or sediment revealed the presence of *V. parahaemolyticus* in over 90% of the samples collected at all time-points throughout the year [36]. Recently, the expansion of fish pathogens into new geographic areas and widening of their host range has been noted, leading to an emergence of new pathogens [33,41]. Contrary to human or animal medicine, fish treatment concerns the entire local population and, therefore, is more difficult to control [33].

## 3. Phage Abundance and Significance in Aquatic Systems

Prior to an investigation of therapeutic phage connotations in the fish and seafood industry, it is important to understand that environmental waters are their natural habitats. Hence, the idea of phage application in aquaculture does not seem to be innovative or groundbreaking. Suffice to say that phage discovery was inseparably associated with waters, and even over 100 years later, this phenomenon is still being investigated in the Ganges [42]. These days we know that in aquatic ecosystems, there is typically a 3- to 10-times greater number of phage particles when compared to their bacterial hosts [43]. Viruses in aquatic habitats are responsible for the mortality of nearly 20–40% prokaryotes every day, affecting community composition and impacting global biogeochemical cycles [44,45]. Despite the small size of phages (approx. 100 nm), they constitute the second-largest biomass in the oceans, just after the prokaryotic biomass [45]. They tend to accumulate in coastal waters rather than in offshore waters, although phages can be found under any latitude, including Arctic sea ice [46]. Analyses of phage genomes revealed not only seasonal phage distribution in waters but also different phage allocations in upper versus deeper water layers [44]. Their existence is shorter in upper layers, possibly due to environmental factors such as sunlight or relatively higher temperatures. Experiments conducted to date have revealed that phages were found in 75% of the water samples collected from environmental habitats and industrial systems, including water treatment plants and drinking water [47].

There is clear evidence that waters have been the most promising source of therapeutic phages since their discovery over 100 years ago [48]. One might assume that the actual known number of phages associated with aquatic environments is significantly larger, as the plethora of their bacterial hosts is difficult to culture in laboratory settings [49]. The situation has changed, thanks to newly developed virome analyses. Without the need to culture both bacteria and phages, scientists can finally become acquainted with a growing number of phage genome sequences. These days, the importance of phages in aquatic environments is of ongoing relevance. When lysing their hosts, viruses cause the release of dissolved organic matter (DOM) in the process called “viral shunt” [50]. Bacteria have a high protein-to-DNA ratio and, therefore, a high nitrogen-to-phosphorus (N:P) ratio. As a result of phage lysis, this larger amount of organic N than organic P is released and made available to other bacteria and phytoplankton for growth. Scientists hypothesize that the effect of lysis on uninfected bacteria production and abundance, as well as on ecosystem functions, including the carbon cycle, might depend on which nutrient (N or P) is limiting. It is estimated that dissolved organic carbon constitutes the largest pool of organic matter in the oceans, and its regulation is a major biogeochemical process. Recently performed studies showed that mutual phage interactions with bacterial hosts are responsible for conversion from organic to inorganic carbon in water environments [51]. In tested samples, the increase in inorganic carbon was 15–25% larger in samples with bacteria and phages when compared to samples with bacteria alone. Notably, the accumulation of inorganic carbon in the form of CO_2_ may have a physiological effect on feed intake, appetite, digestive function, and slowed growth in fish hatcheries. Further, high dissolved CO_2_ levels have been associated with the formation of mineralized deposits in the kidneys of salmonids (nephrocalcinosis), eye cataracts, and increased susceptibility to pathogens [52].

Last but not least, a plethora of phages in water make them ideal candidates to be utilized in other industry sectors. Thanks to their abundance, sometimes restricted to narrow water sources, they can be useful as human-associated fecal indicators [53,54] or in pathogen reduction in wastewater treatment plants [55]. A recent report suggests that marine phages may act as a microbial tracer for the transport of colloidal particles and water flow and contribute to better risk analysis [56].

### Unfavorable Impact of Phages on Aquatic Reservoirs

It is worth noting that the role of phages in spreading antibiotic resistance genes has been raised in a growing number of articles as well, which clearly reflects an increased interest in phage-based therapeutics in general [57,58,59,60]. Metagenomic studies have confirmed the presence of such genes in phages isolated from a vast range of environments, which is in line with their ability to transfer genetic material between hosts. The above referenced vast phage abundance in waters likely contributes to the intensification of the transduction of genes conferring resistance in aquatic niches. In fact, a recent study detected that environmental waters contain a large reservoir of resistance genes, conferring resistance to tetracyclines and β-lactamases. Those genes, detected in phage DNA, were isolated from freshwater, water treatment plants, and oceans [57,59]. Aquatic environments may promote dissemination and acquisition of antibiotic resistance genes [61], which should be taken into consideration in aquacultures. Fish farms, exposed to antibiotic stress, along with high densities of microorganisms and closed water circulation, can provoke horizontal transfer of unwanted genes and the danger of their leakage to the environment [17]. The latest data indicate that antibiotic-resistant bacteria isolated from both aquaculture and agriculture share the same resistance mechanisms, indicating that aquaculture is contributing to the same resistance issues established by agriculture [62]. The presence of remnants of antibiotics and antibiotic resistance genes in major aquacultures in Southeast China has also been pointed out by Chen et al. [63]. Such a phenomenon could be intensified in cases of prolonged, continuous, preventive phage application. In fact, among 41 analyzed genomes of *Aeromonas veronii*, a common human and animal pathogen abundant in aquatic environments, only two strains were free of phage elements [64]. In contrast, there are sources questioning the active role of phages in spreading antibiotic resistance genes in waters. Petrovich et al. [65], in their recent study, could not find strong evidence for phages as vectors for those genes in a hospital wastewater treatment system, suggesting that false-positive samples are possible. One must be aware that the use of antibiotics in fish hatcheries will result in accumulated and unabsorbed antibiotics that are likely to affect phage–host coevolution outside the host [66]. Certainly, the issue of spreading antibiotic resistance is not to be disregarded, and exaggerated optimism should not be a driving force for the extensive use of phages in aquacultures. Antibiotic resistance genes isolated from wastewater samples have been reported to persist longer in phages than in bacteria [67]. Notably, there is a growing number of fish pathogens isolated from aquacultures carrying CRISPR-Cas systems. This pattern was observed mostly in *Flavobacterium* and *Vibrio* species [66]. The more contaminated the waters, the more likely it is that a higher count of microorganisms (including phages and their bacterial hosts) will be found in such samples. Our team has been utilizing this phenomenon for several years during phage isolation. The most valuable samples come from water treatment plants and hospital sewage systems [47]. One could assume that the cumulation of organic matter, chemotherapeutics, and other contaminants in aquacultures could result in a blooming plethora of utterly different phages, with an unexpected impact on environmental balance. Notably, phages have been shown to be resistant, at least to some extent, to chemical agents used in water disinfection and can survive under unfavorable conditions for a long time. We explore this phenomenon further in Section 4.1.

Interestingly, lytic phages may be responsible for biofilm formation [68]. Such an occurrence was noted in marine tunicate *Ciona intestinalis*, whose gut is colonized by *Shewanella*. A study showed that mutual interactions between *Shewanella* and its lytic phages enhanced biofilm formation. Although *Shewanella* is an unusual human pathogen, infections caused by these bacteria have been increasingly accompanied by symptoms such as cellulitis, abscesses, bacteremia, and wound infection [69]. Moreover, prophage induction can also stimulate biofilm formation in *Shewanella* through the release of extracellular DNA, which can be found in the biofilm matrix [68]. 

**Figure 1:**
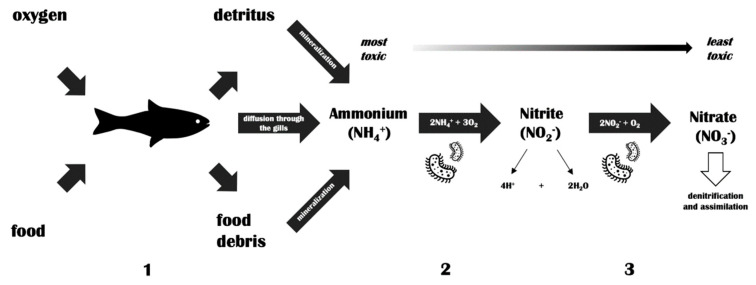
The nitrification pathway in fish tanks. (**1**) Decomposition of organic matter (e.g., fish excrement and uneaten fish food) leads to an increase in ammonium levels in fish tanks. In addition, fish excrete ammonium directly into the water. (**2**) Nitrifying bacteria (*Nitrosomonas*, *Nitrosospira*) oxidize ammonium to nitrite. (**3**) Nitrifying bacteria (*Nitrobacter*, *Nitrospira*) oxidize nitrite to nitrate. The latter is utilized as a plant fertilizer.

Temperate phages were found to act as disrupting agents in the nitrification process, which is one of the most important processes in waters [70]. The authors revealed that the induction of the lytic cycle led to a decrease of the total *Nitrosospira multiformis* count, a bacteria that is responsible for converting ammonia to nitrite. Ammonia is severely toxic and represents an environmental threat for aquatic life, particularly in small reservoirs and at lower pH values. Freshwater fish excrete ammonia directly and immediately into the water and, therefore, its concentrations may rapidly increase in aquacultures of high fish density. Furthermore, dissolved ammonium is colorless and difficult to observe at an early stage of intoxication. As nitrifying bacteria occur together, a rapidly decreasing count of one group could disrupt the entire cycle. However, there is a greater chance for disruption of the nitrogen cycle in water through the use of aquaculture drugs, and phage lytic activity towards nitrifying bacteria should be studied more precisely in terms of possibility, not a real threat. A simplified scheme of the nitrification process is shown in Figure 1.

## 4. Therapeutic Connotations of Phages in Aquacultures

Indisputable differences between natural aquatic niches and aquacultures need to be carefully studied in order to properly conduct phage application. Water parameters such as temperature, salinity, content of dissolved organic matter, pH, and oxygen saturation are subjected to rapid change in small water tanks loaded with different varieties of fish. Obviously, such events should never occur, but small tanks are always at greater risk, especially in systems with high densities of fish, where biomass growth and substrate utilization can be difficult to control. Consequently, spreading disease among fish would be much faster and more lethal in densely-stocked fish tanks. The need to maintain continuously available sources of food to prevent starvation and to promote rapid growth challenges fish workers on a daily basis [71]. A large amount of easy-digestible food increases levels of organic pollutants, such as dioxins and PCBs (polychlorinated biphenyl). A study from 2004 reported on contaminated farmed Atlantic salmon carrying such high levels of PBCs that it could be harmful to humans [72]. All these factors pose a great danger to phage–bacteria interactions through changes in water parameters and may affect the outcome of phage application. Below, we characterize the most crucial aspects that should be considered in aquacultures.

### 4.1. Water Parameters

Studies performed with the use of water samples collected throughout the territory of Poland revealed an unexpected presence of phages in municipal tap water systems [47]. This evidence demonstrates that water disinfection, both chemical and physical, does not significantly affect the lytic activity of at least some phages. Studies performed by Yates et al. [73] revealed that the prevalence and activity of coliphages in groundwaters may be affected by UV irradiation, the presence of organic compounds (particularly, acids from soil humus), and metabolic activity of other aquatic organisms. Interestingly, data collected from numerous water tanks in different parts of the USA showed that pH fluctuations, water clarity, and hardness had no major impact on phage survival. In fact, the only factor inhibiting phage activity was temperature [73]. The importance of temperature for phage lytic activity has been pointed out countless times by several authors over the years [74,75,76,77], along with in-depth molecular analyses of this occurrence. Leon-Velarde et al. [78] revealed that *Yersinia enterocolitica* outer membrane protein OmpF, which acts as a receptor for phages, is subject to strong repression at 37 °C. Thus, the tested phage lysed its host when grown at 25 °C but not at 37 °C. Intriguingly, recent articles imply that temperature may define the outcome of phage–bacteria interactions through the determination of the phage life cycle [79,80]. Switching between cycles possibly regulates the population of the bacterial host throughout the seasons.

Investigations performed by Madsen et al. [81] on *Flavobacterium psychrophilum* phages in salmonid aquacultures have shed more light on the pH-dependent activity of phages. In a laboratory setting, pH had only minor effects on long-term (3 months) phage infectivity within a pH range of 4.5 to 7.5 but inhibited lytic activity below pH 3. Particularly, low pH turned out to be the greatest obstacle in phage attachment to bacterial receptors [36]. Notably, marginal pH values, which proved to be lethal for phages [74,75], are rarely found in environmental waters or fish tanks. Notwithstanding, even in those extreme habitats, phages show unusual adaptation and are profoundly represented in niches of extreme pH, temperatures, and/or salinity. For phages isolated in 2013 from a haloalkaline lake in Kenya, the optimal pH ranges from as high as 10 to 12, values that are not tolerated by most of the lab-cultured phages [82].

Another important factor in terms of phage viability in waters is salinity. Although phages are generally considered more resistant to salinity than bacterial species, osmotic shock has been shown to inactivate phages by even 99% [75]. Very interesting data on phage lytic activity in waters, with possible connotations for fish and seafood industry, come from Silva-Valenzuela et al. [83]. Three *Vibrio cholerae* phages, well-known for their lytic activity in cholera patients in Bangladesh, were not able to infect *V. cholerae* in fresh water. A significant decrease in osmolarity, inorganic nutrients, and carbon sources in the aquatic model caused a loss of bacterial viability and resulted in a lack of phage infection. The authors noted that high salinity was a crucial factor for some *Vibrio* phages’ predation. Salinity in waters is subject to constant change, mostly due to water evaporation at high temperatures. This phenomenon may occur on a much larger scale in fish tanks characterized by limited water capacity, with a possible further impact on the therapeutic outcome of phage treatment. Fluctuations in both viral and prokaryotic heterotrophic production were demonstrated in experimental cross-infections between viruses and prokaryotes from three tropical sites of West Africa, with distinct salinities [84]. The addition of native viruses consistently stimulated viral production. However, the lowest rates of this phenomenon were observed in hypersaline (310‰) water. Choudhury et al. [85] conclude that salinity and pH are two crucial abiotic factors affecting the growth and survival of aquatic organisms. The authors found that the *V. harveyi* phage was active against its host in all three tested salinities, but the highest rate was observed at a salinity of 25 ppt, which was also optimal for the host. A similar analogy was found for the pH. A neutral pH turned out to be the most favorable for both the phage and its host. 

### 4.2. Multiplicity of Infection (MOI)

The ratio of infecting phage particles to bacterial prey is crucial in terms of the effective annihilation of pathogens. Conditions in fish and seafood hatcheries may significantly differ from what we observe in wild waters. Thus, quite a different approach is needed in the context of the therapeutic evaluation of phages in aquatic systems. Typical phage and bacteria densities in waters are often too low for successful phage replication and more favorable conditions are rarely expected to be found in natural water niches [86]. Nilsson et al. [87] observed phage–bacteria dynamics in the Baltic Sea. The number of *Rheinheimera*-infecting phages was strictly correlated to host abundances from June to August between 2012 and 2015. Furthermore, Alonso-Saez et al. [88] found that a high abundance of some marine phages does not imply they are highly active in waters. Our group came to similar conclusions following the testing of *Aeromonas* phages and their bacterial hosts in a small-scale water treatment plant [89]. There was no increase in phage titer after a 24-h incubation period, despite the presence of a specific bacterial host. After several days, we were still able to recover phages introduced to the tank at the beginning of the experiment, which clearly confirms the theory that phages can be resistant to environmental factors, including filtration processes, but they are unable to amplify due to low values of cfu. In conclusion, proper dosing should always be carefully considered before phage application into fish hatcheries. The necessity of high MOI values was pointed out by Kalatzis et al. [21]. Phages against *V. alginolyticus*, an agent responsible for vibriosis in marine aquaculture hatcheries, were effective in vitro only at MOI = 10 and MOI = 100 (a 10- and 100-times higher phage ratio to bacteria, respectively), whereas at MOI = 1, the lytic effect was barely noticeable. Kim et al. [90] used an MOI as high as 10,000 to achieve a protective effect of *Aeromonas salmonicida* phage PAS-1 on rainbow trout. Interestingly, another group revealed quite different results. An in vitro investigation performed on phages against waterborne fish pathogen *Aeromonas salmonicida* showed limited lytic activity at MOI = 10 and almost complete lysis at MOI = 0.01 [91]. Notably, in the first case, the bacterial count started to rise 2 h following phage inoculation but remained at the same low level over the 8 h (end of experiment) at low initial MOI. The authors conclude that heavy phage loads could accelerate the occurrence of bacterial resistance to phages in a relatively short period of time. Such discrepancies require further attention to disentangle complex phage biology in vitro, as well as in vivo, prior to designing phage-based therapeutics. The plethora of interactions was pointed out by Bettarel et al. [84], who observed that viral enrichment in waters, and hence the rise of MOI, elevates chances for contact between phages and bacteria in an environment-dependent manner. Daniels and Wais [92] proposed the theory that a natural environment characterized by a low bacterial load may promote slow phage adsorption to reduce the frequency of release of DNA from phage particles in response to encounters with nonhost material.

Irrespective of MOI values, phages applied in low doses could act as preventive agents; this has been utilized several times for crops [24]. It must be emphasized that our aforementioned experiment was performed without the involvement of fish or other aquatic organisms and, therefore, we were unable to evaluate possible therapeutic connotations in the described model [89]. Studies carried out by Almeida et al. [93] have shed more light on the prophylactic value of phages tested in comparable conditions. The authors used similar phage doses (470 mL of *Flavobacterium* FCL-2 lysate with the titer of 2 × 10^10^ pfu/mL), achieving a final phage density of 1 × 10^7^ pfu/mL in each tested tank with no addition of a bacterial host. Although the authors confirmed that phages recovered during the experiment were derived from the original lysate and not from the environment or natural phage infection, applied doses had a positive impact on the overall health status of the fish. Phages were reisolated from fish mucus and gills, providing a protective effect against *F. columnare* infections. Furthermore, phage addition did not cause negative alterations in water quality or fish health. Prolonged phage persistence in both the described experiments indicates that the protective, rather than therapeutic, effect of phage preparations on fish may be relatively easy to obtain. The absence of lytic activity in *F. psychrophilum* phages challenged with a low bacteria count was presented by Madsen et al. [81]. In the absence of host cells, phage infectivity in pond water decreased by 10,000 times over 55 days. Additionally, the infectivity of the same tested phage decreased over time in a buffer kept at 20 °C, which indicates the necessity of constant supplementation of phage-based preparations in water. The use of phages in the form of probiotics has already been proposed by Soliman et al. [94] as a natural remedy in intensive fish farming.

### 4.3. Fish Immunity

Successful phage therapy can be greatly affected by immune deficiencies of organisms undergoing treatment, which has already been shown on the human model [95,96,97,98]. Furthermore, phages are capable of influencing the immune system of eukaryotes in a variety of ways [99,100]. In aquatic hatcheries, fish are subjected to constant stress due to the extensive use of antibiotics and other drugs and overstocking. Hence, their ability to fight infections may be greatly weakened in different epidemic scenarios. One could predict that phage addition in aquacultures could modulate the immune response and act as an immunostimulant for the induction of antibodies. Silva et al. [101] noted that phage application against *Aeromonas salmonicida* in juvenile Senegalese sole did not impact natural bacterial communities but moderately affected the bacterial community associated with the fish intestinal tract. Evidence for a large amount of naturally occurring phages in the digestive tract of fish was provided by studies performed by He and Yang [102]. In the gut of 62 cultivated freshwater fish, researchers found 63 phages, including vast diversity of *Aeromonas* phages (29), followed by *Vibrio* (1), *Citrobacter* (16), *Serratia* (4), *Enterobacter* (2), *Proteus* (3), *Buttiauxella* (2), *Plesiomonas* (2), *Kluyvera* (1), *Morganella* (2), and *Providencia* (1). The phages contribute to the microbiota balance in the gut ecosystem of fish and modulate their immunity.

The innate immune system consists of barriers limiting the pathogens’ ability to spread throughout the body. The inherent part of the innate immune system is mucus. This is a site of attachment for most pathogens, which causes mucosal infections responsible for high mortality and morbidity among fish [103]. However, phage attachment to mucosal surfaces has been observed as well. Almeida et al. theorize that such binding could create a ubiquitous nonhost-derived immunity against bacterial invaders [103]. As fish are naturally covered by mucus, the authors investigated *Flavobacterium columnare* FCL-2 myophage phage adherence to mucus layers in rainbow trout. Although phage titer in water tanks decreased rapidly below the level of detection, phage particles remained in the mucus of rainbow trout for one week. It is possible that the persistence of FCL-2 phage in the mucus could be the result of the subdiffusive motion created by phage Ig-like folds interacting with mucins, which appears to be stronger than mucus shedding and water flow. Furthermore, the described pretreatment with phages resulted in a delay in disease onset and increased fish survival. The authors also observed increased bacterial susceptibility to phage infection in the mucosal environment. Some authors suggest that phage–bacteria interactions in mucus play a role in lysis–lysogeny switches [66,104]. Whereas the BAM (bacteriophage adherence to mucus) model may promote phages by enabling them to have contact with the host and eliminating potential pathogens from deeper mucus layers, the PtW (piggyback the winner) model may favor bacteria through lysogeny. Interestingly, Barr et al. observed that only binding between phage Ig-like domains and mucin resulted in the reduction of bacterial load, which suggests targeted action rather than accidental movement and attachment [105].

Reports on phage immunomodulatory activity in rainbow trout subjected to phage treatment also came from Schulz et al. [106]. The authors used a commercially available phage cocktail called BAFADOR, intended for use in aquaculture niches. Besides obvious antibacterial action against *Aeromonas hydrophila* and *Pseudomonas fluorescens*, which decreased the mortality of rainbow trout, elevated levels of immunoglobulin, protein, and lysozyme were noted, along with the increased activity of spleen phagocytes and proliferation of pronephros lymphocytes. The same outcome, after the application of BAFADOR, was observed on European eels after experimental challenge with the aforementioned *A. hydrophila* and *P. fluorescens* [107]. These results are consistent with reports of a Chinese group who treated common carp with a phage lysate antigen as a vaccine active against the fish pathogen *A. hydrophila* [108]. The survival rate of fish immunized with the phage vaccine was higher when compared to immunization with formalin-killed bacterial cells six weeks postvaccination. Such a favorable result was accompanied by a robust immune response in the form of higher IL-1β and lysozyme C gene expression, along with higher TNF-α gene expression. In another experiment, rainbow trout, after intramuscular administration of the *Aeromonas salmonicida* phage, showed significant neutralizing properties of its sera at 10 and 15 days postadministration, which declined by 30 days [90]. This sera neutralization did not correspond with phage particles accumulating in kidneys and occurred after the phage was cleared from fish kidneys below the level of detection.

One must be aware that these preliminary reports require follow-up studies on the nonantibacterial action of phages. Nevertheless, reports to date leave hope for the efficient and long-lasting prophylactic effect of phage preparations in fish hatcheries. Interestingly, Laanto et al. [109] proved that phage resistance, developed over time in emerging fish pathogen *F. columnare*, declined bacterial virulence outside the fish host. Thus, prophylactic use of phages could work in three different ways: (a) through direct lytic activity, (b) as immunomodulators in fish tanks through interactions in mucus, and (c) due to phenotypic changes in bacteria that lead to lower bacterial virulence. 

## 5. Phage Application in Fish and Seafood Industry in Practice

A countless number of sources have hypothesized that phages are a promising alternative to antibiotics in industry and medicine. The fish and seafood industry is certainly no exception here. This well-deserved rebirth is a result of numerous more or less analyzed factors, like relatively low cost, safety for the environment and treated organisms, as well as the vast abundance of natural therapeutic agents in the form of phage particles. The latter may be easily investigated and amplified in laboratory settings using well-known and simple techniques. Table 2 presents a summary of recent attempts to control fish and seafood pathogenic bacteria with the use of phage-based preparations. Articles focusing on phage application in aquaculture facilities have skyrocketed in the past decade, which we wanted to emphasize by adding the year of publication to the table. As in-vitro results may differ from what can be achieved in the field, we focused solely on in-vivo experiments conducted over the past decade. Undeniably, one of the major challenges in phage treatment is the use of a single phage versus a phage cocktail [67]. Customizing phage cocktails may be time-consuming, but universal phage therapeutics may not target all desired bacteria. Studies performed by Holmfeldt et al. [110] found a large variation in host range diversity among *Cellulophaga baltica* phages isolated from Swedish and Danish coastal waters. The authors suggest that the well-known narrow host range of marine phages may be the result of inadequate methods for phage isolation (e.g., single-host enrichment). Summarized reports from recent years show the proportional use of both single-phage preparations as well as phage cocktails, along with the use of different MOI ratios. A lack of information regarding applied cfu or pfu in the third column (method of application) means we were unable to obtain such information in some cases.

## 6. Discussion

The phage approach has ignited hope among scientists and fish workers for an imminent breakthrough in the fish and seafood industry, challenged by the abuse of antibiotics and growing demand for high-quality fish products [17]. Notably, the development of other methods in fighting antibiotic resistance, such as bacterial vaccines, has not diminished the importance of a phage approach in any way. In fact, the phage approach is becoming increasingly popular [117]. At first glance, phage treatment appears acceptable and easy to conduct. However, the deeper we investigate this issue, the more complex it appears. Phage administration in aquacultures imposes the most obvious route in the form of water additives or phage-impregnated feed (the latter seems to be more appropriate in prophylactic efforts as infected fish may not take up their food). In every application scenario, preventive or therapeutic, it is important to enable contact between phage particles and bacterial hosts in water, as well as on the surface or inside macroorganisms. Those obstacles should be easily overcome in fish tanks where water circulation is usually maintained by installed pumps or even extensive fish motility in high-stocking tanks. Other obstacles seem to be more challenging. First of all, phage lytic activity in vitro and in vivo may greatly differ as outdoor facilities are exposed to natural fluctuations in physical and chemical water parameters [36]. Such disparity is crucial when biocontrol strategies in the field are taken into account. Comprehensive phage biology, including latency period, burst size, MOI, adsorption rate, lytic spectrum, stability, and host range, should always be evaluated in models mirroring different environmental scenarios.

Selecting phages with a broad host range imposes hurdles in finding a proper one. The majority of all marine phages are highly host-specific. Although there are reports suggesting that selected *Vibrio* phages were able to infect 40% of tested strains, possibly due to the conservative structure of LPS phage receptors [21], the majority of reports indicate vast species diversity in marine niches [36]. Further, Zhang et al. [118] showed that polar flagella in *V. parahaemolyticus*, bacteria inseparably associated with contaminated seafood, can reduce phage infectivity. Notably, such a reduction was not caused by the physical presence of flagella but its rotary movement.

Although phages can decrease bacterial virulence outside fish hosts, some sources imply the opposite. Lysogenic phages have been shown to have the ability to transform nonvirulent bacterial strains into virulent ones [119].

In a review focusing on phage treatment in aquaculture from 2001, the authors mentioned the narrow specificity of phages, which would not harm normal fish intestinal flora, and their self-replicating nature in the presence of susceptible bacteria [120]. These days, scientists are aware that phages can interact with intestinal flora in a way that goes beyond antibacterial action, and their self-replicating nature is noticeable only at a high bacterial count. One must be aware that it is hard to predict how all the abovementioned aspects of phage activity in waters would affect hatcheries when used on a large scale globally. Nevertheless, the pros arising from the use of phages in aquaculture facilities seem to outweigh the cons. Rapid growth and an increasing number of bacterial outbreaks have forced the fish industry to take novel appropriate actions. This was acknowledged by the European Commission, which created and sponsored a special network within the Seventh Framework Programme entitled “Network for the development of phage therapy in aquaculture—AQUAPHAGE” [121]. We can easily assume that the number of such projects will increase in the upcoming years.

## Figures and Tables

**Table 1:** Pathogenic bacterial flora associated with live fish and fish muscle.

Gram-Negative	Gram-Positive
*Escherichia*	*Bacillus*
*Serratia*	*Clostridium*
*Morganella*	*Lactobacillus*
*Vibrio* ^1^	*Corynebacterium*
*Photobacterium* ^1,3^	*Streptococcus* ^6^
*Aeromonas* ^2^	*Renibacterium* ^7^
*Proteus* ^4^	Mycobacteria *
*Alcaligenes* ^5^	
*Enterobacter* ^4^	
*Pseudomonas*	
*Moraxella*	
*Acinetobacter* ^5^	
*Shewanella putrefaciens* ^5^	
*Flavobacterium* ^5^	
*Raoultella*	
*Edwarsiella*	
*Yersinia*	

^1^ Typical of marine waters [18]. ^2^ Typical of fresh waters [18]. ^3^ Found on modified atmosphere-packed salmon [37]. ^4^ Found in spoiled sardine [37]. ^5^ Predominant during the ice storage of fish and shrimp [37]. ^6^ Pathogen of Japanese flounder, rainbow trout, tilapia, and coho salmon [9]. ^7^ Causative agent of bacterial kidney disease affecting salmonid fish [33]. * Described by some authors as neither Gram-positive nor -negative (a Gram stain hardly penetrates the waxy cell wall).

**Table 2:** Phage experiments in vivo conducted on fish and seafood.

Target Bacteria	Fish or Seafood Species	Method of Application	Outcome	Reference (Year)
*Vibrio parahaemolyticus*	Blue mussels *(Mytilus edulus)*	2.5-L glass beakers positioned at 4 °C with 8–16 infected (10^9^ cfu/mL) mussels and approx. 0.1 × 10^6^ pfu of phage cocktail (12 phages) added prior to experiment	Phage cocktail was effective in significantly reducing *V. parahaemolyticus* to undetectable numbers in mussels	[36] (2018)
*Vibrio parahaemolyticus*	Shrimp (*Penaeus vannamei*)	Infected (5.0 × 10^5^ cfu/mL) juvenile shrimps were fed with pellets containing the phage (1.5 × 10^8^ pfu/shrimp) or immersed in phage suspension (1.5 × 10^6^ pfu/mL) 1 h after the bacterial challenge or prior to infection	Mortality in groups treated 1 h after bacterial infection was 100%; prophylactic use of phages resulted in mortality varied from 25% to 50%	[13] (2017)
*Streptococcus agalactiae*	Nile tilapia *(Oreochromis niloticus)*	Single phage preparation added to fish tanks	Treated fish had 60% survival rates and a delayed mean death time of about three days when compared to the control group	[111] (2018)
*Aeromonas hydrophila*	Loach *(Misgurnus anguillicaudatus)*	Infected (1 × 10^7^ cfu/mL) loah treated by immersion in water containing 1.0 × 10^8^ pfu/mL of single phage preparation	Mortality rates were 16%, 53%, 57%, and 56.67% after 24, 48, 72, 96 h respectively when compared to the control group with 100% mortality; most of the surviving fish showed no disease symptoms	[112] (2020)
*Vibrio anguillarum*	Atlantic cod (Gadus morhua)	Infected (0.5–1 × 10^6^ cfu/mL) fish eggs were incubated at 5.5 °C (Atlantic cod) and 15.5 °C (turbot) in 24-well plates with 2 mL sterile and oxygenated seawater with addition of single phage preparation to a final concentration of 0.5–8 × 10^8^ pfu/mL	The maximum reduction in mortality varied from 29% to 92% for turbot and from 49% to 86% for Atlantic cod assessed during the experiment and depending on the strain used; notably, reduction in mortality was not significant in the majority of cases at the end of the experiment	[113] (2018)
*Vibrio* sp. VA-F3	Shrimp *(Litopenaeus vannamei)*	30 infected (2 × 10^6^ cfu/mL) shrimps received the treatment of phage cocktail (5 phages) at 2 × 10^7^ pfu/mL	Survival rate assessed after seven days of cultivation reached 91.4% when compared to 20% rate in the untreated control group	[114] (2019)
*Flavobacterium psychrophilum*	*Salmo salar*, *Oncorhynchus mykiss*	Fish were infected by intraperitoneal injection of bacteria and single phage mixture at MOI = 10 pfu/cfu and were kept at 15 °C for 15 days	Percentage mortality reduction in the presence of the phage varied from 16% to 100%	[115] (2012)
*Vibrio splendidus*	Sea cucumber (*Apostichopus japonicus*)	Diet supplemented with three phages alone or as a cocktail was implemented for 60 days before immersion in seawater with 6 × 10^6^ cfu/mL of bacterial pathogen	Survival rate during the next ten days was 18% for the control group, 82% for the phage cocktail, and 65%, 58%, 50% for the three phages applied alone	[16] (2016)
*Vibrio harveyi*	Black tiger shrimp (*Litopenaeus monodon*)	Shrimp postlarvae (PL2 stage) were acclimated for three days in 1.25-L glass flasks. Next, 10^10^ pfu/mL single phage were added and 30 min later 10^7^ cfu/mL *V. harveyi*	After 10 days, mortality in the treated group was 20% when compared to >70% in tanks challenged only with *V. harveyi*	[15] (2014)
*Vibrio parahaemolyticus*	Oysters	Oysters infected with multidrug-resistant pandemic strain were immersed in solution containing single phage	After 72 h bacterial growth reduction was from 8.9 × 10^6^ cfu/mL (control group) to 1.94 cfu/mL (treatment group)	[116] (2014)
*Aeromonas salmonicida subsp. salmonicida*	Rainbow trout (*Oncorhynchus mykiss*)	3–4-month rainbow trout were kept in aerated 50 L glass tanks (20 fish/tank). Fish were intramuscularly injected with 2.5 × 10^2^ cfu/fish and with single phage at MOI = 10,000 immediately after the bacterial challenge; fish were observed for 14 days	Fish in the treated group showed a 26.7% survival rate; the surviving fish did not show ulcerative lesions and remained healthy until 14 days postadministration; all fish from the control group died	[90] (2012)
*Aeromonas salmonicida*	Senegalese sole (*Solea senegalensis*)	Infected Senegalese sole juveniles were treated with single phage preparation	After 72 h, infected fish juveniles treated with phages showed no mortality contrary to 36% mortality in the control group	[101] (2016)

## References

[1] FAO (2020). The State of World Fisheries and Aquaculture 2020. Sustainability in Action.

[2] (2020). Overview of the Seafood Industry. https://www.seafoodhealthfacts.org/seafood-choices/overview-seafood-industry.

[3] Swanson D., Block R., Mousa S. (2012). Omega-3 Fatty Acids EPA and DHA: Health Benefits Throughout Life1. Adv. Nutr..

[4] Whittle P. (2020). Seafood Industry Struggling to Stay Afloat amid Outbreak. AP News. https://apnews.com/308352a4521171c83284a850bb892277.

[5] Cabello F.C., Godfrey H.P., Tomova A., Ivanova L., Dölz H., Millanao A., Buschmann A.H. (2013). Antimicrobial use in aquaculture re-examined: Its relevance to antimicrobial resistance and to animal and human health. Environ. Microbiol..

[6] Endersen L., O’Mahony J., Hill C., Ross R.P., McAuliffe O., Coffey A. (2014). Phage Therapy in the Food Industry. Annu. Rev. Food Sci. Technol..

[7] Institute of Food Technologists (2006). Antimicrobial Resistance: Implications for the Food System. An Expert Report, Funded by the IFT Foundation. Compr. Rev. Food Sci. Food Saf..

[8] Liu X., Steele J.C., Meng X.-Z. (2017). Usage, residue, and human health risk of antibiotics in Chinese aquaculture: A review. Environ. Pollut..

[9] Richards G.P. (2014). Bacteriophage remediation of bacterial pathogens in aquaculture: A review of the technology. Bacteriophage.

[10] Yu Y.-P., Gong T., Jost G., Liu W.-H., Ye D.-Z., Luo Z.-H. (2013). Isolation and characterization of five lytic bacteriophages infecting a Vibrio strain closely related to Vibrio owensii. FEMS Microbiol. Lett..

[11] Chu W.-H., Zhu W. (2009). Isolation of Bdellovibrio as Biological Therapeutic Agents Used For the Treatment of Aeromonas hydrophila Infection in Fish. Zoonoses Public Health.

[12] Kim J.H., Gomez D.K., Nakai T., Park S.C. (2010). Isolation and identification of bacteriophages infecting ayu Plecoglossus altivelis altivelis specific Flavobacterium psychrophilum. Vet. Microbiol..

[13] Jun J.W., Han J.E., Giri S.S., Tang K.F., Zhou X., Aranguren L.F., Kim H.J., Yun S., Chi C., Park S.C. (2017). Phage Application for the Protection from Acute Hepatopancreatic Necrosis Disease (AHPND) in Penaeus vannamei. Indian J. Microbiol..

[14] Stalin N., Srinivasan P. (2017). Efficacy of potential phage cocktails against Vibrio harveyi and closely related Vibrio species isolated from shrimp aquaculture environment in the south east coast of India. Vet. Microbiol..

[15] Patil J.R., Desai S.N., Roy P., Durgaiah M., Saravanan R.S., Vipra A. (2014). Simulated hatchery system to assess bacteriophage efficacy against Vibrio harveyi. Dis. Aquat. Org..

[16] Li Z., Li X., Zhang J., Wang X., Wang L., Cao Z., Xu Y. (2016). Use of phages to control Vibrio splendidus infection in the juvenile sea cucumber Apostichopus japonicus. Fish Shellfish Immunol..

[17] Miranda C.D., Godoy F., Lee M.R. (2018). Current Status of the Use of Antibiotics and the Antimicrobial Resistance in the Chilean Salmon Farms. Front. Microbiol..

[18] Huss H.H. (1995). FAO Fisheries Technical Paper—348.

[19] FAO (2016). The State of World Fisheries and Aquaculture 2016: Contributing to Food Security and Nutrition for All.

[20] Leung T.L.F., Bates A.E. (2012). More rapid and severe disease outbreaks for aquaculture at the tropics: Implications for food security. J. Appl. Ecol..

[21] Kalatzis P.G., Bastías R., Kokkari C., Katharios P. (2016). Isolation and Characterization of Two Lytic Bacteriophages, φSt2 and φGrn1; Phage Therapy Application for Biological Control of Vibrio alginolyticus in Aquaculture Live Feeds. PLoS ONE.

[22] De Melo A.G., Levesque S., Moineau S. (2018). Phages as friends and enemies in food processing. Curr. Opin. Biotechnol..

[23] Komora N., Bruschi C., Ferreira V., Maciel C., Brandão T.R., Fernandes R., Saraiva J.A., Castro S.M., Teixeira P. (2018). The protective effect of food matrices on Listeria lytic bacteriophage P100 application towards high pressure processing. Food Microbiol..

[24] Żaczek M., Weber-Dąbrowska B., Górski A. (2014). Phages in the global fruit and vegetable industry. J. Appl. Microbiol..

[25] García P., Martinez B., Obeso J., Rodríguez A. (2008). Bacteriophages and their application in food safety. Lett. Appl. Microbiol..

[26] Kosznik-Kwaśnicka K., Topka G., Dydecka A., Necel A., Nejman-Faleńczyk B., Bloch S., Węgrzyn G., Węgrzyn A., Górski A., Międzybrodzki R., Borysowski J. (2019). The Use of Bacteriophages in Animal Health and Food Protection. Phage Therapy: A Practical Approach.

[27] Strauch E., Hammerl J.A., Hertwig S. (2015). Bacteriophages: New Tools for Safer Food?. Rev. Chil. Infectol..

[28] Katznelson H. (1937). Bacteriophage in relation to plant diseases. Bot. Rev..

[29] Miguéis S., Saraiva C., Esteves A. (2017). Efficacy of LISTEX P100 at Different Concentrations for Reduction of Listeria monocytogenes Inoculated in Sashimi. J. Food Prot..

[30] Soni K.A., Nannapaneni R., Hagens S. (2010). Reduction of Listeria Monocytogenes on the Surface of Fresh Channel Catfish Fillets by Bacteriophage Listex P100. Foodborne Pathog. Dis..

[31] Zhang X., Niu Y.D., Nan Y., Stanford K., Holley R., McAllister T.A., Narvaez-Bravo C. (2019). SalmoFresh™ effectiveness in controlling Salmonella on romaine lettuce, mung bean sprouts and seeds. Int. J. Food Microbiol..

[32] Vikram A., Tokman J.I., Woolston J., Sulakvelidze A. (2020). Phage Biocontrol Improves Food Safety by Significantly Reducing the Level and Prevalence of Escherichia coli O157:H7 in Various Foods. J. Food Prot..

[33] Sudheesh P.S., Al-Ghabshi A., Al-Mazrooei N., Al-Habsi S. (2012). Comparative Pathogenomics of Bacteria Causing Infectious Diseases in Fish. Int. J. Evol. Biol..

[34] Rostami H., Abbaszadeh S., Shokri S. (2017). Combined effects of lactoperoxidase system-whey protein coating and modified atmosphere packaging on the microbiological, chemical and sensory attributes of Pike-Perch fillets. J. Food Sci. Technol..

[35] Erkmen O., Bozoglu T.F., Erkmen O., Bozoglu T.F. (2016). Spoilage of Fish and Other Seafoods. Chapter 18. Food Microbiology: Principles into Practice.

[36] Onarinde B.A., Dixon R.A. (2018). Prospects for Biocontrol of Vibrio parahaemolyticus Contamination in Blue Mussels (Mytilus edulus)—A Year-Long Study. Front. Microbiol..

[37] Kuley E., Durmuş M., Balikci E., Uçar Y., Regenstein J.M., Özogul F. (2016). Fish spoilage bacterial growth and their biogenic amine accumulation: Inhibitory effects of olive by-products. Int. J. Food Prop..

[38] Gram L., Huss H.H. (1996). Microbiological spoilage of fish and fish products. Int. J. Food Microbiol..

[39] Froelich B., Noble R.T. (2016). Vibrio bacteria in raw oysters: Managing risks to human health. Philos. Trans. R. Soc. B Biol. Sci..

[40] Haq S.M., Dayal H.H. (2005). Chronic Liver Disease and Consumption of Raw Oysters: A Potentially Lethal Combination—A Review of Vibrio vulnificus Septicemia. Am. J. Gastroenterol..

[41] Austin B., Austin D.A. (2016). Bacterial Fish Pathogens. Disease of Farmed and Wild Fish.

[42] Dwivedi S., Chauhan P.S., Mishra S., Kumar A., Singh P.K., Kamthan M., Chauhan R., Awasthi S., Yadav S., Mishra A. (2020). Self-cleansing properties of Ganga during mass ritualistic bathing on Maha-Kumbh. Environ. Monit. Assess..

[43] Weinbauer M.G. (2004). Ecology of prokaryotic viruses. FEMS Microbiol. Rev..

[44] Kavagutti V.S., Andrei A.Ş., Mehrshad M., Salcher M.M., Ghai R. (2019). Phage-centric ecological interactions in aquatic ecosystems revealed through ultra-deep metagenomics. Microbiome.

[45] Breitbart M. (2012). Marine Viruses: Truth or Dare. Annu. Rev. Mar. Sci..

[46] Yu Z.-C., Chen X.-L., Shen Q.-T., Zhao D.-L., Tang B.-L., Su H.-N., Wu Z.-Y., Qin Q.-L., Xie B.-B., Zhang X.-Y. (2015). Filamentous phages prevalent in Pseudoalteromonas spp. confer properties advantageous to host survival in Arctic sea ice. ISME J..

[47] Weber-Dąbrowska B., Żaczek M., Dziedzic B., Łusiak-Szelachowska M., Kiejzik M., Górski A., Gworek B., Wierzbicki K., Eymontt A., Méndez-Vilas A. (2014). Bacteriophages in green biotechnology—The utilization of drinking water. Industrial, Medical and Environmental Applications of Microorganisms: Current Status and Trends.

[48] Adhya S., Merril C. (2006). The road to phage therapy. Nature.

[49] Moon K., Kang I., Kim S., Kim S.-J., Cho J.-C. (2018). Genomic and ecological study of two distinctive freshwater bacteriophages infecting a Comamonadaceae bacterium. Sci. Rep..

[50] Pourtois J., Tarnita C.E., Bonachela J.A. (2020). Impact of Lytic Phages on Phosphorus- vs. Nitrogen-Limited Marine Microbes. Front. Microbiol..

[51] Sanmukh S., Khairnar K., Paunikar W., Lokhande S. (2015). Understanding carbon regulation in aquatic systems—Bacteriophages as a model. F1000Research.

[52] Skov P.V. (2019). CO2 in aquaculture. The Cardiovascular System—Development, Plasticity and Physiological Responses.

[53] McMINN B.R., Korajkic A., Ashbolt N. (2014). Evaluation ofBacteroides fragilis GB-124 bacteriophages as novel human-associated faecal indicators in the United States. Lett. Appl. Microbiol..

[54] Blanch A.R., Lucena F., Muniesa M., Jofre J. (2020). Fast and easy methods for the detection of coliphages. J. Microbiol. Methods.

[55] Periasamy D., Sundaram A. (2013). A novel approach for pathogen reduction in wastewater treatment. J. Environ. Health Sci. Eng..

[56] Ghanem N., Kiesel B., Kallies R., Harms H., Chatzinotas A., Wick L.Y. (2016). Marine Phages As Tracers: Effects of Size, Morphology, and Physico–Chemical Surface Properties on Transport in a Porous Medium. Environ. Sci. Technol..

[57] Balcazar J.L. (2018). How do bacteriophages promote antibiotic resistance in the environment?. Clin. Microbiol. Infect..

[58] Muniesa M., Colomer-Lluch M., Jofre J. (2013). Potential impact of environmental bacteriophages in spreading antibiotic resistance genes. Future Microbiol..

[59] Moon K., Jeon J.H., Kang I., Park K.S., Lee K., Cha C.-J., Lee S.H., Cho J.-C. (2020). Freshwater viral metagenome reveals novel and functional phage-borne antibiotic resistance genes. Microbiome.

[60] Brown-Jaque M., Calero-Cáceres W., Muniesa M. (2015). Transfer of antibiotic-resistance genes via phage-related mobile elements. Plasmid.

[61] Marti E., Variatza E., Balcázar J.L. (2014). The role of aquatic ecosystems as reservoirs of antibiotic resistance. Trends Microbiol..

[62] Done H.Y., Venkatesan A.K., Halden R.U. (2015). Does the Recent Growth of Aquaculture Create Antibiotic Resistance Threats Different from those Associated with Land Animal Production in Agriculture?. AAPS J..

[63] Chen C., Zheng L., Zhou J., Zhao H. (2017). Persistence and risk of antibiotic residues and antibiotic resistance genes in major mariculture sites in Southeast China. Sci. Total Environ..

[64] Tekedar H.C., Kumru S., Blom J., Perkins A.D., Griffin M.J., Abdelhamed H., Karsi A., Lawrence M.L. (2019). Comparative genomics of Aeromonas veronii: Identification of a pathotype impacting aquaculture globally. PLoS ONE.

[65] Petrovich M.L., Zilberman A., Kaplan A., Eliraz G.R., Wang Y., Langenfeld K., Duhaime M., Wigginton K., Poretsky R., Avisar D. (2020). Microbial and Viral Communities and Their Antibiotic Resistance Genes Throughout a Hospital Wastewater Treatment System. Front. Microbiol..

[66] Hoikkala V., Almeida G.M.D.F., Laanto E., Sundberg L.-R. (2019). Aquaculture as a source of empirical evidence for coevolution between CRISPR-Cas and phage. Philos. Trans. R. Soc. B Biol. Sci..

[67] Lin D.M., Koskella B., Lin H.C. (2017). Phage therapy: An alternative to antibiotics in the age of multi-drug resistance. World J. Gastrointest. Pharm..

[68] Middelboe M., Brussaard C.P.D. (2017). Marine Viruses: Key Players in Marine Ecosystems. Viruses.

[69] Sharma K.K., Kalawat U. (2010). Emerging Infections: Shewanella—A Series of Five Cases. J. Lab. Physicians.

[70] Batinovic S., Wassef F., Knowler S.A., Rice D.T.F., Stanton C.R., Rose J., Tucci J., Nittami T., Vinh A., Drummond G.R. (2019). Bacteriophages in Natural and Artificial Environments. Pathogens.

[71] Allen P.J., Steeby J.A. (2011). Aquaculture: Challenges and Promise. Nat. Educ. Knowl..

[72] Hites R.A., A Foran J., Carpenter D.O., Hamilton M.C., Knuth B.A., Schwager S.J. (2004). Global Assessment of Organic Contaminants in Farmed Salmon. Science.

[73] Yates M.V., Gerba C.P., Kelley L.M. (1985). Virus persistence in groundwater. Appl. Environ. Microbiol..

[74] Jończyk-Matysiak E., Łodej N., Kula D., Owczarek B., Orwat F., Międzybrodzki R., Neuberg J., Bagińska N., Weber-Dąbrowska B., Górski A. (2019). Factors determining phage stability/activity: Challenges in practical phage application. Expert Rev. Anti-Infect..

[75] Jończyk E., Kłak M., Międzybrodzki R., Górski A. (2011). The influence of external factors on bacteriophages—Review. Folia Microbiol..

[76] Kim J.-W., Kathariou S. (2009). Temperature-Dependent Phage Resistance of Listeria monocytogenes Epidemic Clone II. Appl. Environ. Microbiol..

[77] Groman N.B., Suzuki G. (1962). Temperature and Lambda Phage Reproduction. J. Bacteriol..

[78] Leon-Velarde C.G., Happonen L., Pajunen M.I., Leskinen K., Kropinski A.M., Mattinen L., Rajtor M., Zur J., Smith D., Chen S. (2016). Yersinia enterocolitica-Specific Infection by Bacteriophages TG1 and ϕR1-RT Is Dependent on Temperature-Regulated Expression of the Phage Host Receptor OmpF. Appl. Environ. Microbiol..

[79] Shan J., Korbsrisate S., Withatanung P., Adler N.R.L., Clokie M.R.J., Galyov E.E. (2014). Temperature dependent bacteriophages of a tropical bacterial pathogen. Front. Microbiol..

[80] Egilmez H.I., Morozov A., Clokie M.R.J., Shan J., Letarov A., Galyov E.E. (2018). Temperature-dependent virus lifecycle choices may reveal and predict facets of the biology of opportunistic pathogenic bacteria. Sci. Rep..

[81] Madsen L., Bertelsen S.K., Dalsgaard I., Middelboe M. (2013). Dispersal and Survival of Flavobacterium psychrophilum Phages In Vivo in Rainbow Trout and In Vitro under Laboratory Conditions: Implications for Their Use in Phage Therapy. Appl. Environ. Microbiol..

[82] Akhwale J.K., Rohde M., Rohde C., Bunk B., Spröer C., Boga H.I., Klenk H.-P., Wittmann J. (2019). Isolation, characterization and analysis of bacteriophages from the haloalkaline lake Elmenteita, Kenya. PLoS ONE.

[83] Silva C., Camilli A. (2019). Niche adaptation limits bacteriophage predation of Vibrio cholerae in a nutrient-poor aquatic environment. Proc. Natl. Acad. Sci. USA.

[84] Bettarel Y., Desnues A., Rochelle-Newall E. (2010). Lytic failure in cross-inoculationassays between phages and prokaryotes fromthree aquatic sites of contrasting salinity. FEMS Microbiol. Lett..

[85] Choudhury T.G., Maiti B., Venugopal M.N., Karunasagar I. (2019). Influence of some environmental variables and addition of r-lysozyme on efficacy of Vibrio harveyi phage for therapy. J. Biosci..

[86] Muniesa M., Jofre J. (2004). Factors influencing the replication of somatic coliphages in the water environment. Antonie Leeuwenhoek.

[87] Nilsson E., Li K., Fridlund J., Šulčius S., Bunse C., Karlsson C.M.G., Lindh M., Lundin D., Pinhassi J., Holmfeldt K. (2019). Genomic and Seasonal Variations among Aquatic Phages Infecting the Baltic Sea Gammaproteobacterium Rheinheimera sp. Strain BAL341. Appl. Environ. Microbiol..

[88] Alonso-Sáez L., Morán X.A.G., Clokie M.R. (2018). Low activity of lytic pelagiphages in coastal marine waters. ISME J..

[89] Wierzbicki K. (2020). Potencjalna Technologia Biologicznej Stabilizacji Mikrobiologii Wody Przeznaczonej do Spożycia.

[90] Kim J.H., Choresca C.H., Shin S.P., Han J.E., Jun J.W., Park S.C. (2013). Biological Control ofAeromonas salmonicidasubsp.salmonicidaInfection in Rainbow Trout (Oncorhynchus mykiss) UsingAeromonasPhage PAS-1. Transbound. Emerg. Dis..

[91] Chen L., Yuan S., Liu Q., Mai G., Yang J., Deng D., Zhang B., Liu C., Ma Y. (2018). In Vitro Design and Evaluation of Phage Cocktails Against Aeromonas salmonicida. Front. Microbiol..

[92] Daniels L.L., Wais A.C. (1998). Virulence in phage populations infecting Halobacterium cutirubrum. FEMS Microbiol. Ecol..

[93] Almeida G.M.D.F., Mäkelä K., Laanto E., Pulkkinen J., Vielma J., Sundberg L.-R. (2019). The Fate of Bacteriophages in Recirculating Aquaculture Systems (RAS)—Towards Developing Phage Therapy for RAS. Antibiotics.

[94] Soliman W.S., Shaapan R.M., Mohamed L.A., Gayed S.S. (2019). Recent biocontrol measures for fish bacterial diseases, in particular to probiotics, bio-encapsulated vaccines, and phage therapy. Open Vet. J..

[95] Międzybrodzki R., Borysowski J., Weber-Dąbrowska B., Fortuna W., Letkiewicz S., Szufnarowski K., Pawełczyk Z., Rogóż P., Kłak M., Wojtasik E. (2012). Clinical Aspects of Phage Therapy. Adv. Appl. Microbiol..

[96] Łusiak-Szelachowska M., Żaczek M., Weber-Dąbrowska B., Międzybrodzki R., Kłak M., Fortuna W., Letkiewicz S., Rogóż P., Szufnarowski K., Jonczyk-Matysiak E. (2014). Phage Neutralization by Sera of Patients Receiving Phage Therapy. Viral Immunol..

[97] Żaczek M., Łusiak-Szelachowska M., Weber-Dąbrowska B., Międzybrodzki R., Fortuna W., Rogóż P., Letkiewicz S., Górski A., Górski A., Międzybrodzki R., Borysowski J. (2019). Humoral Immune Response to Phage-Based Therapeutics. Phage Therapy: A Practical Approach.

[98] Żaczek M., Łusiak-Szelachowska M., Jończyk-Matysiak E., Weber-Dąbrowska B., Międzybrodzki R., Owczarek B., Kopciuch A., Fortuna W., Rogóż P., Górski A. (2016). Antibody Production in Response to Staphylococcal MS-1 Phage Cocktail in Patients Undergoing Phage Therapy. Front. Microbiol..

[99] Górski A., Dąbrowska K., Międzybrodzki R., Weber-Dąbrowska B., Łusiak-Szelachowska M., Jończyk-Matysiak E., Borysowski J. (2017). Phages and immunomodulation. Future Microbiol..

[100] Górski A., Międzybrodzki R., Jończyk-Matysiak E., Żaczek M., Borysowski J. (2019). Phage-specific diverse effects of bacterial viruses on the immune system. Future Microbiol..

[101] Silva Y.J., Moreirinha C., Pereira C., Costa L., Rocha R.J.M., Cunha Â., Gomes N., Calado R., Almeida M.A. (2016). Biological control of Aeromonas salmonicida infection in juvenile Senegalese sole (Solea senegalensis) with Phage AS-A. Aquaculture.

[102] He Y., Yang H. (2014). The gastrointestinal phage communities of the cultivated freshwater fishes. FEMS Microbiol. Lett..

[103] Almeida G.M.D.F., Laanto E., Ashrafi R., Sundberg L.-R. (2019). Bacteriophage Adherence to Mucus Mediates Preventive Protection against Pathogenic Bacteria. MBio.

[104] Silveira C.B., Rohwer F. (2016). Piggyback-the-Winner in host-associated microbial communities. npj Biofilms Microbiomes.

[105] Barr J.J., Auro R., Sam-Soon N., Kassegne S., Peters G., Bonilla N., Hatay M., Mourtada S., Bailey B., Youle M. (2015). Subdiffusive motion of bacteriophage in mucosal surfaces increases the frequency of bacterial encounters. Proc. Natl. Acad. Sci. USA.

[106] Schulz P., Pajdak J., Robak S., Dastych J., Siwicki A.K. (2019). Bacteriophage-based cocktail modulates selected immunological parameters and post-challenge survival of rainbow trout (Oncorhynchus mykiss). J. Fish Dis..

[107] Schulz P., Robak S., Dastych J., Siwicki A.K. (2019). Influence of bacteriophages cocktail on European eel (Anguilla anguilla) immunity and survival after experimental challenge. Fish Shellfish Immunol..

[108] Yun S., Jun J.W., Giri S.S., Kim H.J., Chi C., Kim S.G., Kim S.W., Kang J.W., Han S.J., Kwon J. (2019). Immunostimulation of Cyprinus carpio using phage lysate of Aeromonas hydrophila. Fish Shellfish Immunol..

[109] Laanto E., Bamford J.K.H., Laakso J., Sundberg L.-R. (2012). Phage-Driven Loss of Virulence in a Fish Pathogenic Bacterium. PLoS ONE.

[110] Holmfeldt K., Middelboe M., Nybroe O., Riemann L. (2007). Large Variabilities in Host Strain Susceptibility and Phage Host Range Govern Interactions between Lytic Marine Phages and Their Flavobacterium Hosts. Appl. Environ. Microbiol..

[111] Luo L., Liao G., Liu C., Jiang X., Lin M., Zhao C., Tao J., Huang Z. (2018). Characterization of bacteriophage HN48 and its protective effects in Nile tilapia Oreochromis niloticus against Streptococcus agalactiae infections. J. Fish Dis..

[112] Akmal M., Rahimi-Midani A., Hafeez-Ur-Rehman M., Hussain A., Choi T.-J. (2020). Isolation, Characterization, and Application of a Bacteriophage Infecting the Fish Pathogen Aeromonas hydrophila. Pathogens.

[113] Rørbo N., Rønneseth A., Kalatzis P.G., Rasmussen B.B., Engell-Sørensen K., Kleppen H.P., Wergeland H.I., Gram L., Middelboe M. (2018). Exploring the Effect of Phage Therapy in Preventing Vibrio anguillarum Infections in Cod and Turbot Larvae. Antibiotics.

[114] Chen L., Fan J., Yan T., Liu Q., Yuan S., Zhang H., Yang J., Deng D., Huang S., Ma Y. (2019). Isolation and Characterization of Specific Phages to Prepare a Cocktail Preventing Vibrio sp. Va-F3 Infections in Shrimp (Litopenaeus vannamei). Front. Microbiol..

[115] Castillo D., Higuera G., Villa M., Middelboe M., Dalsgaard I., Madsen L., Espejo R. (2012). Diversity of Flavobacterium psychrophilum and the potential use of its phages for protection against bacterial cold water disease in salmonids. J. Fish Dis..

[116] Jun J.W., Kim H.J., Kil Yun S., Chai J.Y., Park S.C. (2014). Eating oysters without risk of vibriosis: Application of a bacteriophage against Vibrio parahaemolyticus in oysters. Int. J. Food Microbiol..

[117] Pirnay J.-P., De Vos D., Verbeken G. (2019). Clinical application of bacteriophages in Europe. Microbiol. Aust..

[118] Zhang H., Li L., Zhao Z., Peng D., Zhou X. (2016). Polar flagella rotation in Vibrio parahaemolyticus confers resistance to bacteriophage infection. Sci. Rep..

[119] Hai N. (2015). The use of probiotics in aquaculture. J. Appl. Microbiol..

[120] Nakai T., Park S.C. (2002). Bacteriophage therapy of infectious diseases in aquaculture. Res. Microbiol..

[121] The Seventh Framework Programme—AQUAPHAGE. https://cordis.europa.eu/project/id/269175.

